# ^18^F–FDG-PET/CT and diffusion-weighted MRI for monitoring a BRAF and CDK 4/6 inhibitor combination therapy in a murine model of human melanoma

**DOI:** 10.1186/s40644-018-0135-y

**Published:** 2018-01-18

**Authors:** Ralf S. Eschbach, Philipp M. Kazmierczak, Maurice M. Heimer, Andrei Todica, Heidrun Hirner-Eppeneder, Moritz J. Schneider, Georg Keinrath, Olga Solyanik, Jessica Olivier, Wolfgang G. Kunz, Maximilian F. Reiser, Peter Bartenstein, Jens Ricke, Clemens C. Cyran

**Affiliations:** 1 Department of Radiology, Laboratory for Experimental Radiology, University Hospital, Ludwig-Maximilians-University Munich, Marchioninistr. 15, 81377 München, Germany; 2 Department of Nuclear Medicine, University Hospital, Ludwig-Maximilians-University Munich, Marchioninistr. 15, 81377 München, Germany; 3 Comprehensive Pneumology Center, German Center for Lung Research, Munich, Germany

**Keywords:** Melanoma, BRAF inhibitor, CDK inhibitor, ^18^F–FDG-PET, Diffusion-weighted MRI, Therapy monitoring

## Abstract

**Background:**

The purpose of the study was to investigate a novel BRAF and CDK 4/6 inhibitor combination therapy in a murine model of BRAF-V600-mutant human melanoma monitored by ^18^F–FDG-PET/CT and diffusion-weighted MRI (DW-MRI).

**Methods:**

Human BRAF-V600-mutant melanoma (A375) xenograft-bearing balb/c nude mice (*n* = 21) were imaged by ^18^F–FDG-PET/CT and DW-MRI before (day 0) and after (day 7) a 1-week BRAF and CDK 4/6 inhibitor combination therapy (*n* = 12; dabrafenib, 20 mg/kg/d; ribociclib, 100 mg/kg/d) or placebo (*n* = 9). Animals were scanned on a small animal PET after intravenous administration of 20 MBq ^18^F–FDG. Tumor glucose uptake was calculated as the tumor-to-liver-ratio (TTL). Unenhanced CT data sets were subsequently acquired for anatomic coregistration. Tumor diffusivity was assessed by DW-MRI using the apparent diffusion coefficient (ADC). Anti-tumor therapy effects were assessed by *ex vivo* immunohistochemistry for validation purposes (microvascular density – CD31; tumor cell proliferation – Ki-67).

**Results:**

Tumor glucose uptake was significantly suppressed under therapy (∆TTL_Therapy_ − 1.00 ± 0.53 vs. ∆TTL_Control_ 0.85 ± 1.21; *p* < 0.001). In addition, tumor diffusivity was significantly elevated following the BRAF and CDK 4/6 inhibitor combination therapy (∆ADC_Therapy_ 0.12 ± 0.14 × 10^−3^ mm^2^/s; ∆ADC_Control_ − 0.12 ± 0.06 × 10^−3^ mm^2^/s; *p* < 0.001). Immunohistochemistry revealed a significant suppression of microvascular density (CD31, 147 ± 48 vs. 287 ± 92; *p* = 0.001) and proliferation (Ki-67, 3718 ± 998 vs. 5389 ± 1332; *p* = 0.007) in the therapy compared to the control group.

**Conclusion:**

A novel BRAF and CDK 4/6 inhibitor combination therapy exhibited significant anti-angiogenic and anti-proliferative effects in experimental human melanomas, monitored by ^18^F–FDG-PET/CT and DW-MRI.

## Background

The mitogen-activated protein kinase (MAPK) pathway controls cell cycle progression, survival, and proliferation in human cells [1, 2]. B-rapidly accelerated fibrosarcoma (BRAF) gene mutations V600E/K were identified as key drivers of oncogenesis in melanoma, as they lead to overactivation of the MAPK pathway and uncontrolled cell proliferation [2]. Thus, selective inhibition of the BRAF gene emerged as a novel, targeted treatment regimen in advanced or unresectable melanoma [3, 4]. However, tumor response to BRAF inhibitor monotherapy may be limited by intrinsic or acquired resistance [5–7]. Consequently, combination therapies of BRAF inhibitors and additional therapeutics potentially overcoming resistance were investigated in recent years [5, 8]. One possible mechanism of acquired BRAF inhibitor resistance is MAPK pathway activation via the mitogen-activated extracellular signal-regulated kinase (MEK). Co-targeting the MAPK pathway by a BRAF and MEK inhibitor combination therapy yields high response rates as well as prolonged overall and progression-free survival in advanced BRAF-mutant melanoma compared to other available treatment strategies, including BRAF inhibitor monotherapy [9]. A possible mechanism of intrinsic BRAF inhibitor resistance is cyclin D1 overexpression with cyclin-dependent kinase (CDK) 4 mutation/amplification [2, 5]. Cyclin D1 promotes cell cycle progression by binding to CDK 4 and 6, which act as downstream mediators of the MAPK pathway and regulate the cell cycle via the retinoblastoma tumor suppressor protein [2, 5]. Yadav et al. recently demonstrated that the selective inhibition of CDK 4/6 leads to tumor growth regression in a BRAF inhibitor-resistant *in vivo* model of human melanoma (A375) [10]. In 2017, based on the results of the MONALEESA-2 trial, the U.S. Food and Drug Administration approved the CDK 4/6 inhibitor ribociclib combined with an aromatase inhibitor for the treatment of advanced or metastatic, hormone receptor-positive and human epidermal growth factor receptor 2-negative breast cancer in postmenopausal women [11]. The addition of a CDK 4/6 inhibitor to BRAF inhibitor monotherapy represents a novel strategy to overcome cyclin D1-dependent resistance. Dual inhibition of the MAPK pathway by a BRAF and CDK 4/6 inhibitor combination therapy may thus be a promising future therapy regimen in advanced melanoma.

Imaging plays a central role for the non-invasive tumor response assessment in clinical oncology. Morphology-based criteria of tumor response, e. g., RECIST (Response Evaluation Criteria in Solid Tumors), provide a valuable clinical tool to differentiate between partial/complete response, progressive, and stable disease [12]. These criteria are based on the number and size of tumor manifestations, which are commonly assessed by morphological imaging modalities such as computed tomography (CT) or magnetic resonance imaging (MRI). However, in contrast to traditional, primarily cytotoxic therapies, novel targeted therapies exhibit only subtle effects on tumor size [13]. Thus, morphology-based tumor response criteria are of only limited applicability in targeted therapy regimens [14]. Functional and molecular imaging modalities allow for a non-invasive tumor characterization beyond morphology, delivering information on tumor pathophysiology such as tumor glucose metabolism (^18^F–fluorodeoxyglucose positron emission tomography; ^18^F–FDG-PET) and tumor cellularity (diffusion-weighted MRI; DW-MRI). Both ^18^F–FDG-PET and DW-MRI demonstrated their potential to generate non-invasive imaging biomarkers of therapy response in melanoma under targeted therapy [15–18].

As a proof of principle, the present study is a first approach to explore a selective CDK 4/6 inhibitor as novel combination compound for dual inhibition of the MAPK signal pathway in melanoma therapy. The aim of this experimental study was to close this gap of knowledge, evaluating a novel BRAF and CDK 4/6 inhibitor combination therapy in a murine model of human BRAF-V600-mutant melanoma using a multimodal imaging protocol of ^18^F–FDG-PET/CT and DW-MRI. We hypothesized that a BRAF and CDK 4/6 inhibitor combination therapy exhibits significant anti-angiogenic and anti-proliferative effects in experimental human melanomas in mice and that the according alterations in tumor pathophysiology can be non-invasively monitored by ^18^F–FDG-PET/CT and DW-MRI *in vivo* validated by *ex vivo* immunohistochemistry.

## Methods

The experiments were performed in accordance with the Guidelines for the Care and Use of Laboratory Animals of the National Institutes of Health and with approval by the Government Committee for Animal Research.

### Animal model and experimental protocol

**Fig. 1:**
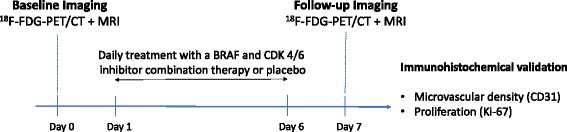
Experimental protocol. ^18^F–FDG-PET/CT and MRI baseline scans were performed on day 0. Animals received either a BRAF and CDK4/6 inhibitor combination therapy (therapy group) or a volume-equivalent placebo (control group) administered daily over the course of 6 days. Follow-up ^18^F–FDG-PET/CT and MRI scans were performed on day 7. Subsequent to follow-up imaging, animals were sacrificed. The tumors were explanted to undergo the immunohistochemical analysis with regard to tumor microvascular density (CD31) and tumor cell proliferation (Ki-67)

After diluting human melanoma cells (A375, ATCC® CRL-1619™, CLS Cell Lines Service GmbH, Eppelheim, Germany) in a total volume of 0.1 mL as a 1:1 solution of phosphate buffered saline (PBS pH 7.4; GIBCO Life Technologies, Darmstadt, Germany) and Matrigel™ (BD Biosciences, San Jose, CA), 3 × 10^6^ cells per mouse were injected subcutaneously into the left abdominal flank of *n* = 21 athymic balb/c nude mice (Charles River, Sulzfeld, Germany). When tumors reached a diameter of 0.5 cm, animals were randomly assigned to either the therapy (*n* = 12) or the control group (*n* = 9). Imaging was performed on day 0 (baseline) and day 7 (follow-up) using a multimodal imaging protocol of ^18^F–FDG PET/CT and DW-MRI. Imaging was performed under inhalation anesthesia (2.5% in 1.0 L 100% O_2_/min for induction, 1.5% in 1.0 L 100% O_2_/min for maintenance). Subsequently to the baseline scan, animals were treated daily with either a combination of BRAF inhibitor dabrafenib (20 mg/kg/day; Novartis AG, Basel, Switzerland) and CDK4/6 inhibitor ribociclib (100 mg/kg/day; Novartis AG, Basel, Switzerland) in the therapy group or a volume-equivalent placebo solution (0.5% hydroxymethyl cellulose and 0.2% Tween-80 in ddH_2_0) in the control group. Immediately after follow-up imaging on day 7, animals were sacrificed and tumors were explanted and fixed in formalin for immunohistochemical analysis with regard to tumor microvascular density (CD31) and tumor cell proliferation (Ki-67). Figure 1 provides an overview of the experimental protocol.

### PET imaging

Small-animal PET was performed on a preclinical PET scanner (Inveon μPET, Siemens Healthineers, Erlangen, Germany). For hygienic reasons and allowing regulated anesthesia delivery, mice were placed inside a custom-built acrylic glass-imaging chamber in prone position. 45 min after manual intravenous injection of 20 MBq (~100 μL) ^18^F–FDG per animal, a transmission scan (15 min) was acquired for scatter and attenuation correction. Subsequently, PET list-mode data acquisition was initiated from 60 to 90 min (30 min emission).

**Fig. 2:**
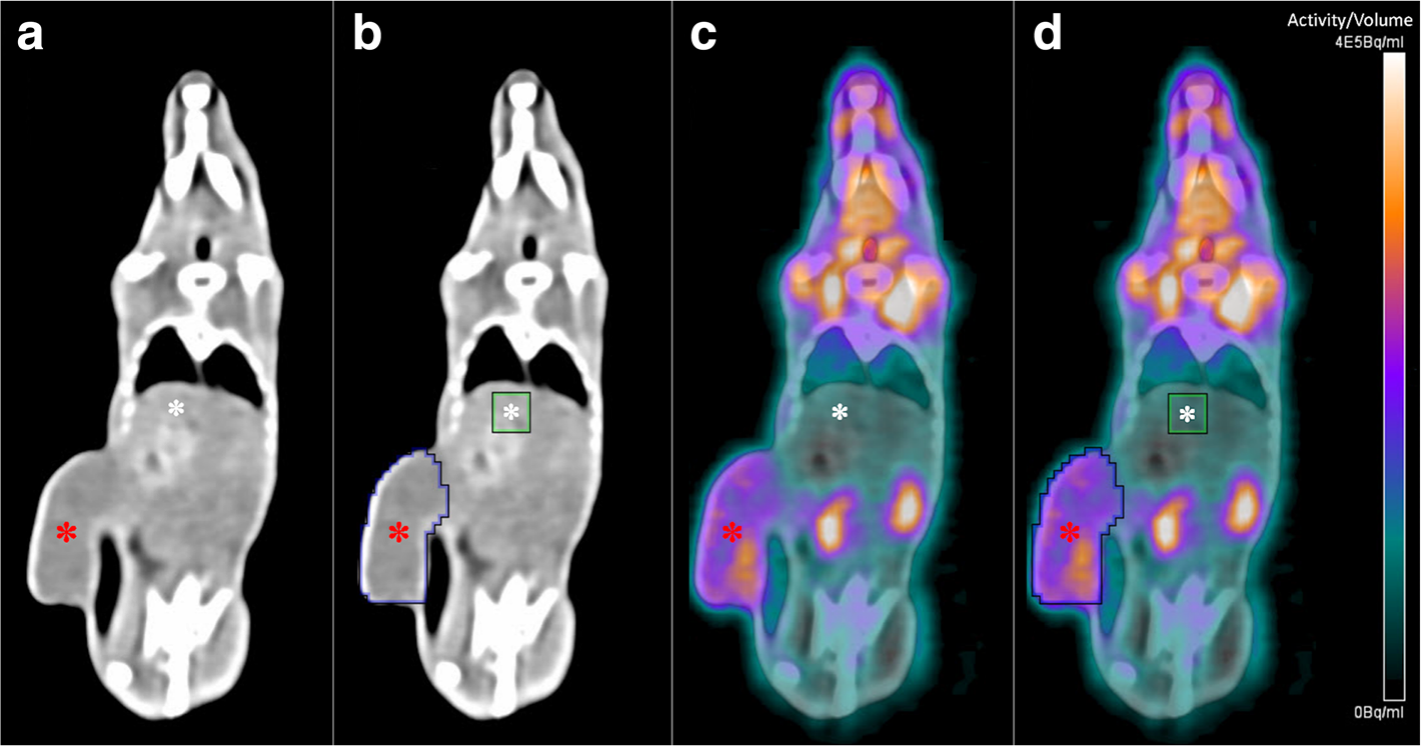
PET/CT VOI Selection. **a** and **b**: coronal unenhanced CT without (**a**) and with (**b**) VOIs. **c** and **d**: Fused coronal PET/CT data sets without (**c**) and with (**d**) VOIs. Note the tumor in the lateral flank (red asterisk). The liver is indicated by a white asterisk. To calculate the metabolic tumor volume, a PET/CT-guided VOI surrounding the tumor was drawn. Background was determined in a VOI in the right liver lobe and the TTL was calculated accordingly

All PET data were post-processed on an Inveon Acquisition Workplace (Siemens Healthineers, Erlangen, Germany). Images were reconstructed as a static image using OSEM 3D (4 iterations) and MAP 3D (32 iterations) algorithm in a 256 × 256 matrix with a zoom value of 100% as described previously [19]. To calculate the metabolic tumor volume (MTV), a PET/CT-guided volume of interest (VOI) surrounding the tumor was drawn manually and a threshold-value of 30% of the hottest voxels was applied. Background was determined as mean uptake in a 9 mm^3^ VOI in the right liver lobe. Tumor-to-liver-ratio (TTL, VOImaxtumor/VOImeanliver) was determined as a semiquantitative measurement of tumor radiotracer accumulation before (day 0) and after treatment (day 7). Figure 2 demonstrates the process of PET/CT VOI selection.

After PET measurements, additional CT images were acquired on a latest generation clinical CT (SOMATOM Force, Siemens Healthineers, Erlangen, Germany), maintaining the animals in the same position within the acrylic imaging chamber for morphological correlation, as described previously [19].

### MRI

MRI measurements were conducted on a clinical 3 Tesla scanner (MAGNETOM Skyra, Siemens Healthineers, Erlangen, Germany) using a 16-channel wrist coil (Siemens Healthineers, Erlangen, Germany). Mice were positioned head-first in prone position. For the assessment of tumor morphology and tumor volume as morphology-based surrogate of tumor response, T2-weighted MR images were acquired using a 2D Turbo Spin Echo sequence (TR = 5470 ms, TE = 91 ms, in-plane resolution 0.3 × 0.3 mm, matrix size 192 × 192, slice thickness 1.5 mm). In addition, DW-MRI data sets were acquired using a single shot echo planar imaging sequence (TR = 3200 ms, TE = 50 ms, FOV = 84 mm, acquisition matrix = 64 × 64, reconstructed matrix = 128 × 128, slice thickness 2 mm, 8 averages, time to acquisition 180 s; 3 b-values: b = 0 s/mm^2^/200 s/mm^2^/800 s/mm^2^). Apparent diffusion coefficient (ADC) maps were calculated by voxel-wise least-squares fitting of the model

$$ \mathrm{S}\left(\mathrm{b}\right)={\mathrm{S}}_0\cdotp {\mathrm{e}}^{-\mathrm{b}\cdotp \mathrm{ADC}}. $$

MRI data sets were analyzed on an external workstation using a dedicated, in-house written post-processing software (PMI; Platform for Research in Medical Imaging, version 0.4) [20]. For each measurement, a multi-slice VOI was drawn manually over the entire tumor. The median value inside this VOI was used to calculate a representative tumor ADC value for statistical analysis.

### Immunohistochemistry

Three micrometer tissue sections were cut from the formalin-fixed and paraffin-embedded tumor tissue and stained with regard to microvascular density (CD31) and tumor cell proliferation (Ki-67). After de-waxing and rehydration following standard procedures (pre-heating at 60 °C, washing in xylene substitute (Neo-Clear, Merck KgaA, Darmstadt, Germany) and rehydration in a graded series of ethanol (100, 95, 80, and 70%, respectively) followed by double distilled water, antigen demasking was performed by microwave irradiation at 600 W in a 0.1 M citrate buffer solution (pH 6.0). Tissue samples were subsequently antibody-incubated at 4 °C over night (antibodies: monoclonal rabbit anti-Ki-67 antibody; SP6, Abcam ab16667 1:100, Cambridge, United Kingdom; polyclonal rabbit anti-CD31 primary antibody; Abcam ab28364 1:50, Cambridge, United Kingdom).

According to the manufacturer’s instructions, further work-up of tissue samples was performed using the EnVision + System HRP (DAB or AEC) (DAKO Diagnostika, Hamburg, Germany) kit. Slides were counterstained using Mayer’s Haemalaun (Merck KgaA, Darmstadt, Germany) and covered with Kaiser’s Glycerin Gelatine (Merck KgaA, Darmstadt, Germany). Results were quantified as the number of positively stained nuclei (Ki-67) or positively stained microvessels (CD31) in ten random fields at 200× magnification.

### Statistical analysis

In this randomized placebo-controlled preclinical trial the statistical analyses were performed using commercially available statistics software (SPSS 23, IBM Corp., Armonk, NY). For intergroup comparisons of the imaging and the immunohistochemical parameters, the Mann-Whitney-U-test was applied. For intragroup comparisons between baseline (day 0) and follow-up (day 7), a Wilcoxon signed-rank test was performed. Correlations between the imaging and the immunohistochemical parameters were assessed by Spearman’s correlation coefficient. Continuous variables were presented as means with standard deviations. The confirmatory tests were performed against a significance level α = 0.05 with Bonferroni corrections.

## Results

The experimental protocol was successfully completed in *n* = 21 animals. There were no significant (*p* > 0.05) intergroup differences between therapy and control group in baseline TTL (TTL_Baseline_: 4.19 ± 0.97 vs. 3.70 ± 0.90; *p* = 0.193) and baseline ADC (ADC_Baseline_: 0.78 ± 0.10 × 10^−3^ mm^2^/s vs. 0.79 ± 0.10 × 10^−3^ mm^2^/s; *p* = 0.754) as well as in metabolic (161.1 ± 86.5 mm^3^ vs. 217.9 ± 145.8 mm^3^; *p* = 0.464) and morphological (94.6 ± 71.4 mm^3^ vs. 134.3 ± 89.0 mm^3^; *p* = 0.219) tumor volumes.

### ^18^F–FDG-PET/CT

**Fig. 3:**
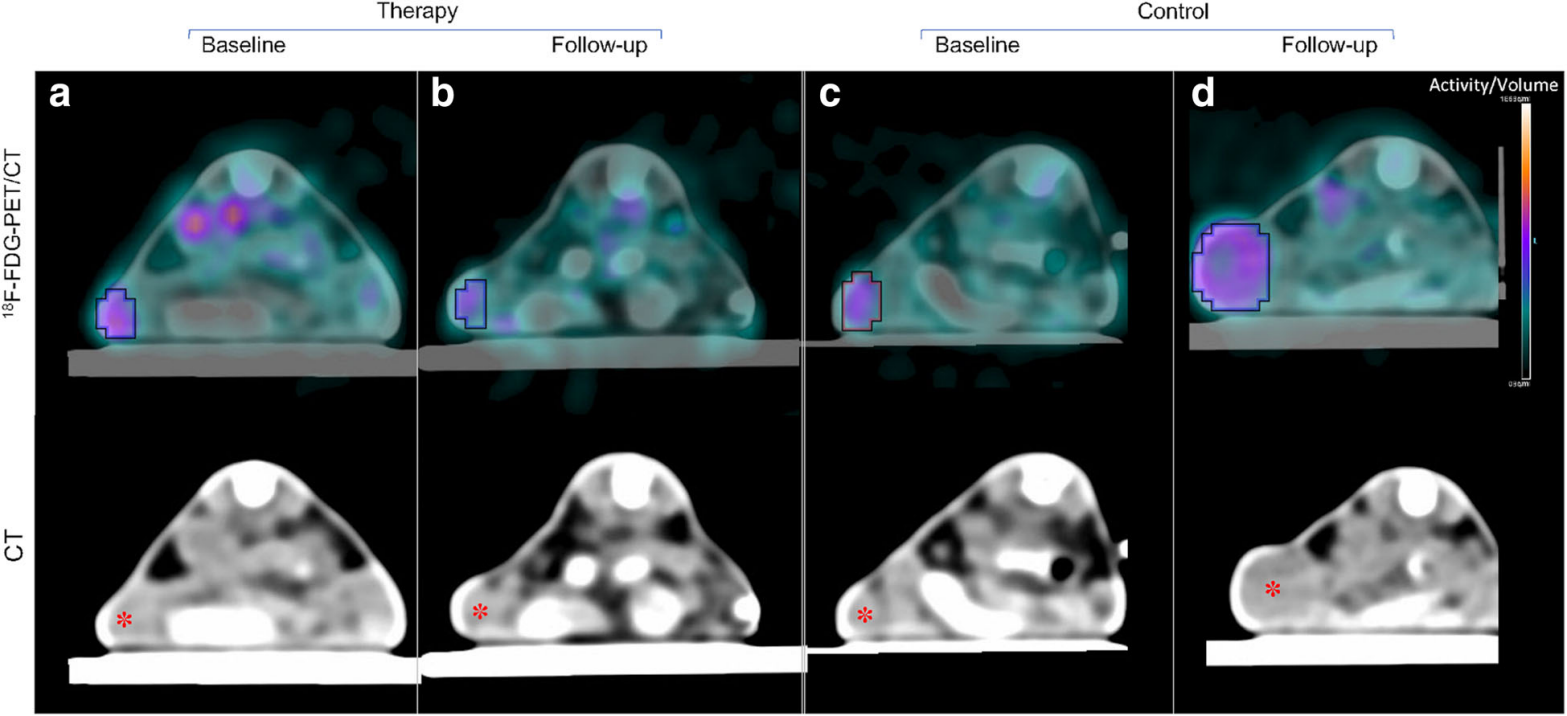
Axial PET/CT data sets of representative tumors from therapy and control group. Top row: ^18^F–FDG-PET/CT data sets. Bottom row: CT data sets. **a** therapy group, baseline. **b** therapy group, follow-up. **c** control group, baseline. **d** control group, follow-up. Note the significantly suppressed tumor glucose uptake following the 1-week BRAF and CDK 4/6 inhibitor combination therapy (**b** vs. **a**). Note the significant increase in tumor glucose uptake and tumor volume in the control group (**d** vs. **c**)

We observed a significant reduction of TTL under therapy (TTL_Therapy_ from 4.19 ± 0.97 to 3.19 ± 0.97; *p* = 0.002). In the control group, TTL demonstrated a non-significant increase from baseline to follow-up (TTL_Control_ from 3.70 ± 0.90 to 4.55 ± 0.91; *p* = 0.14). Follow-up TTL values were significantly lower in the therapy group (TTL_Follow-up_: 3.19 ± 0.97 vs. 4.55 ± 0.91; *p* = 0.007). Moreover, ∆TTL (TTL_Follow-up_ – TTL_Baseline_) was negative and significantly lower in the therapy compared to the control group (∆TTL_Therapy_ − 1.00 ± 0.53 vs. ∆TTL_Control_ 0.85 ± 1.21; *p* < 0.001). The metabolic tumor volume increased in the control group (from 217.9 ± 145.8 mm^3^ on day 0 to 573.5 ± 294.7 mm^3^ on day 7; *p* = 0.008), while no significant change was observed in the therapy group (from 161.1 ± 86.5 mm^3^ to 211.6 ± 139.4 mm^3^; *p* = 0.308). Metabolic tumor volumes on follow-up were significantly higher in the control than in the therapy group (573.5 ± 294.7 mm^3^ vs. 211.6 ± 139.4 mm^3^; *p* = 0.001). Figure 3 shows representative ^18^F–FDG-PET/CT data sets from the therapy and the control group. Individual TTL values and metabolic tumor volumes at baseline and at follow-up are provided in Table 1.

**Table 1:** Individual values for tumor glucose uptake and metabolic tumor volumes at baseline and follow-up

Number	Group^a^	TTL_Baseline_	TTL_Follow-Up_	∆TTL	Volume_Baseline_ *(mm*^*3*^*)*	Volume_Follow-up_ *(mm*^*3*^*)*	∆Volume *(mm*^*3*^*)*
1	T	5.63	4.83	−0.79	296.0	311.4	15.4
2	T	4.80	3.13	−1.67	86.8	50.4	−36.4
3	T	5.90	5.26	−0.64	56.1	162.7	106.6
4	T	3.72	2.65	−1.07	155.9	417.9	262.0
5	T	3.94	3.88	0.06	142.5	433.7	291.2
6	T	4.57	3.02	−1.55	230.8	185.2	−45.6
7	T	4.61	2.90	−1.71	112.8	78.2	−34.6
8	T	3.69	2.40	−1.29	162.7	118.0	−44.7
9	T	3.11	2.52	−0.59	332.0	392.0	60.0
10	T	3.59	2.46	−1.13	171.3	161.7	−9.6
11	T	4.14	2.93	−1.21	120.0	80.6	−39.4
12	T	2.57	2.29	−0.28	66.2	147.8	81.6
Mean ± SD^b^	T	4.19 ± 0.97	3.19 ± 0.97	−1.00 ± 0.53	161.1 ± 86.5	211.6 ± 139.4	50.5 ± 117.9
13	C	4.34	4.06	−0.28	96.9	344.0	247.1
14	C	3.07	5.13	2.06	519.1	1192.3	673.2
15	C	5.62	5.50	−0.12	213.0	616.1	403.1
16	C	2.88	3.95	1.07	140.1	329.1	189.0
17	C	4.02	4.13	0.11	222.6	597.8	375.2
18	C	3.31	5.25	1.94	165.1	511.0	345.9
19	C	3.50	5.45	1.95	115.6	421.3	305.7
20	C	2.71	4.73	2.02	392.5	866.5	474.0
21	C	3.81	2.72	−1.09	96.0	283.1	187.1
Mean ± SD^b^	C	3.70 ± 0.90	4.55 ± 0.91	0.85 ± 1.21	217.9 ± 145.8	573.5 ± 294.7	355.6 ± 153.3

^a^T = therapy group; C = control group

^b^SD = standard deviation

### MRI

**Fig. 4:**
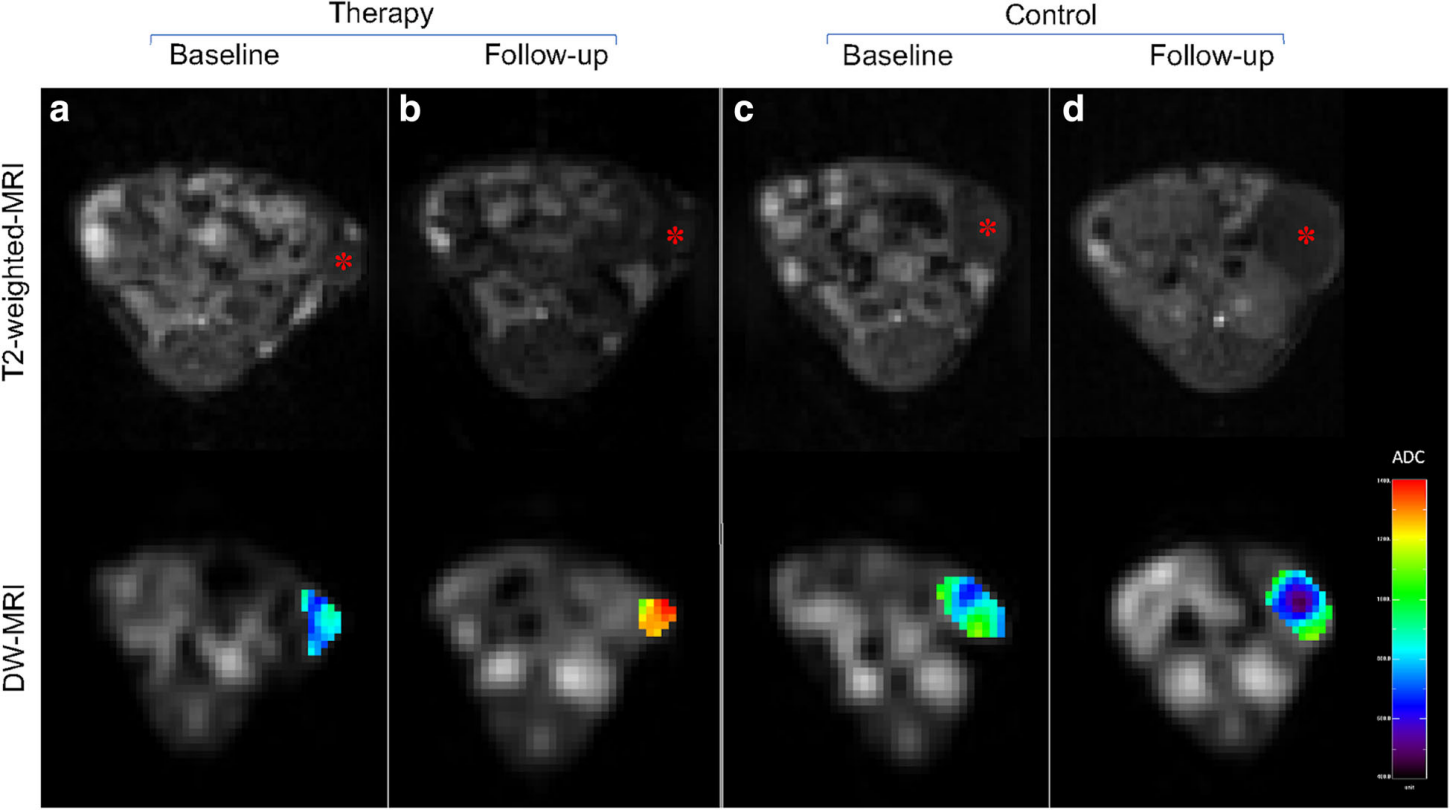
Axial MRI data sets of representative tumors from therapy and control group. Top row: T2-weighted MRI data sets. Bottom row: DW-MRI data sets. **a**: therapy group, baseline. **b**: therapy group, follow-up. **c**: control group, baseline. **d**: control group, follow-up. Note the significantly increased tumor diffusivity under therapy (**b** vs. **a**) and the significantly decreased tumor diffusivity in the control group (**d** vs. **c**)

**Fig. 5:**
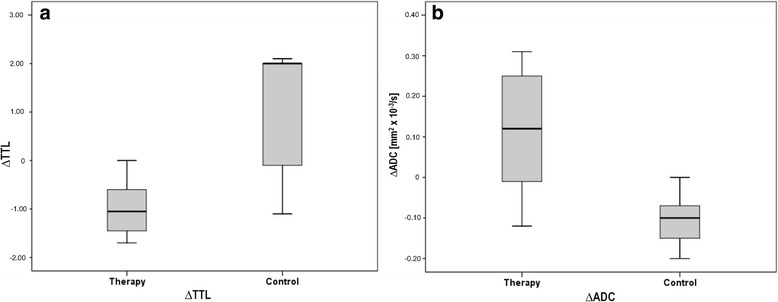
∆TTL and ∆ADC values in therapy and control group. Note the significant suppression of tumor glucose metabolism (**a**) as well as the significant increase of tumor diffusivity under therapy (**b**)

DW-MRI detected an increase in ADC under therapy with a trend toward significance (ADC_Therapy_: from 0.78 ± 0.10 × 10^−3^ mm^2^/s to 0.90 ± 0.13 × 10^−3^ mm^2^/s; *p* = 0.026). In the control group, the ADC showed a significant decline (ADC_Control_: from 0.79 ± 0.10 × 10^−3^ mm^2^/s to 0.68 ± 0.06 × 10^−3^ mm^2^/s; *p* = 0.012). Follow-up ADC values were significantly higher in the therapy group (0.90 ± 0.13 × 10^−3^ mm^2^/s vs. 0.68 ± 0.06 × 10^−3^ mm^2^/s in the control group; *p* < 0.001). Moreover, ∆ADC (ADC_Follow-up_ – ADC_Baseline_) was significantly elevated in the therapy group (∆ADC_Therapy_ 0.12 ± 0.14 × 10^−3^ mm^2^/s; ∆ADC_Control_ − 0.12 ± 0.06 × 10^−3^ mm^2^/s; *p* < 0.001). Analogously to the metabolic tumor volumes, the morphological tumor volumes measured on T2w images significantly increased in the control group (from 134.3 ± 89.0 mm^3^ to 381.9 ± 179.4 mm^3^; *p* = 0.008). In the therapy group, the morphological tumor volumes showed a non-significant increase between baseline and follow-up (from 94.6 ± 71.4 mm^3^ to 130.8 ± 91.3 mm^3^; *p* = 0.071). Figure 4 shows representative MRI data sets from the therapy and the control group. Table 2 displays individual ADC values and the morphological tumor volumes for the therapy and the control group. Figure 5 shows boxplot diagrams of ∆TTL and ∆ADC for the therapy and the control group.

**Table 2:** Individual values for tumor diffusivity and morphological tumor volumes at baseline and follow-up

Number	Group^a^	ADC_Baseline_ *(mm*^*2*^*x10*^*−3*^*/s)*	ADC_Follow-up_ *(mm*^*2*^*x10*^*−3*^*/s)*	∆ADC *(mm*^*2*^*x10*^*−3*^*/s)*	Volume_Baseline_ *(mm*^*3*^*)*	Volume_Follow-up_ *(mm*^*3*^*)*	∆Volume *(mm*^*3*^*)*
1	T	0.84	0.84	0.00	163.0	166.3	3.3
2	T	0.88	1.16	0.28	25.7	33.5	7.8
3	T	0.74	0.72	−0.02	19.1	99.5	80.4
4	T	0.77	0.86	0.09	188.8	255.5	66.7
5	T	0.87	0.82	−0.05	88.5	281.1	192.6
6	T	0.62	0.84	0.22	154.9	107.7	−47.2
7	T	0.79	1.07	0.28	67.2	48.3	−18.9
8	T	0.71	0.87	0.16	38.7	50.8	12.1
9	T	0.66	0.83	0.17	232.5	264.1	31.6
10	T	0.73	0.81	0.08	62.0	123.0	61.0
11	T	0.76	1.07	0.31	48.9	31.9	−17.0
12	T	0.97	0.85	−0.12	45.6	107.9	62.3
Mean ± SD^b^	T	0.78 ± 0.10	0.90 ± 0.13	0.12 ± 0.14	94.6 ± 71.4	130.8 ± 91.3	36.2 ± 63.2
13	C	0.85	0.77	−0.08	119.0	356.0	237.0
14	C	0.70	0.63	−0.07	320.6	777.6	457.0
15	C	0.86	0.69	−0.17	103.1	379.7	276.6
16	C	0.96	0.76	−0.20	81.5	212.9	131.4
17	C	0.78	0.63	−0.15	138.0	371.4	233.4
18	C	0.73	0.61	−0.12	109.0	292.9	183.9
19	C	0.87	0.71	−0.16	72.7	301.7	229.0
20	C	0.61	0.61	0.00	232.7	541.0	308.3
21	C	0.79	0.69	−0.10	31.8	203.5	171.7
Mean ± SD^b^	C	0.79 ± 0.10	0.68 ± 0.06	−0.12 ± 0.06	134.3 ± 89.0	381.9 ± 179.4	247.6 ± 95.1

^a^T = therapy group; C = control group

^b^SD = standard deviation

### Immunohistochemistry

**Fig. 6:**
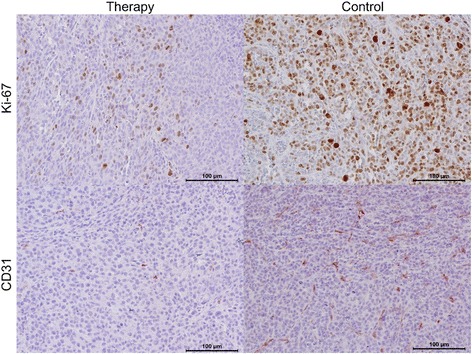
Tumor cell proliferation and microvascular density in representative tumor sections. Left column: therapy. Right column: control. Top row: proliferation (Ki-67). Bottom row: microvascular density (CD31). Note the significantly lower tumor proliferation (Ki-67) and tumor microvascular density (CD31) in the therapy compared to the control group

Multiparametric immunohistochemistry revealed a significantly lower microvascular density (CD31: 147 ± 48 vs. 287 ± 92; *p* = 0.001) and tumor cell proliferation (Ki-67: 3718 ± 998 vs. 5389 ± 1332; *p* = 0.007) in the therapy compared to the control group. Table 3 summarizes individual immunohistochemical values for both groups. Figure 6 shows representative tumor sections from the therapy and the control group.

**Table 3:** Individual immunohistochemical parameters

Number	Group^a^	CD31	Ki-67
1	T	166	4433
2	T	83	5831
3	T	212	3189
4	T	129	2649
5	T	222	2832
6	T	182	3820
7	T	79	4033
8	T	105	2912
9	T	100	2227
10	T	151	4306
11	T	179	4210
12	T	151	4177
Mean ± SD^b^	T	147 ± 48	3718 ± 998
13	C	271	6801
14	C	336	6349
15	C	447	6489
16	C	191	3799
17	C	307	4562
18	C	358	4582
19	C	261	3379
20	C	284	5718
21	C	132	6824
Mean ± SD^b^	C	287 ± 92	5389 ± 1332

^a^T = therapy group; C = control group

^b^SD = standard deviation

### Correlations between imaging and immunohistochemical parameters

TTL demonstrated a good, significant correlation to microvascular density (CD31, ρ = 0.79; *p* < 0.001) and no significant correlation to the proliferation rate (Ki-67, ρ = 0.33; *p* = 0.14). ADC showed a strong and highly significant inverse correlation to microvascular density (CD31, ρ = −0.80; *p* < 0.001) but no significant correlation to proliferation (Ki-67, ρ = −0.42; *p* = 0.061). ∆TTL and ∆ADC showed strong and significant inverse correlations (ρ = −0.75; *p* = 0.002).

## Discussion

In the present study, we investigated ^18^F–FDG-PET/CT and DW-MRI for the *in vivo* monitoring of a novel BRAF and CDK4/6 inhibitor combination therapy in human melanoma xenografts in mice. Dual inhibition of the MAPK signal pathway demonstrated significant anti-angiogenic and anti-proliferative effects in the investigated tumor model. The multimodal imaging protocol allowed for the *in vivo* monitoring of tumor glucose metabolism and tumor diffusivity, adding molecular and functional information to the established morphology-based assessments of tumor response.

Our results are in line with previous preclinical and clinical studies investigating tumor response to targeted MAPK signal pathway inhibition. Baudy et al. reported a reduction in tumor glucose metabolism in A375 xenografts in mice following a BRAF (vemurafenib) and MEK inhibitor (GDC-0973) combination therapy over the course of 6 days [21]. However, the authors validated the imaging results by tumor cell glucose transporter 1 and MAPK pathway protein expression but not by immunohistochemical markers of microvascular density or tumor cell proliferation. Analogously, combined BRAF and MEK targeting (vemurafenib plus cobimetinib or dabrafenib plus trametinib) lead to a significant reduction in tumor maximum standardized uptake value (SUVmax) in patients with advanced melanoma with a mean time to follow-up of 26 days [22]. ^18^F–FDG-PET even provided predictive imaging biomarkers of therapy response in the investigated patient population, with a significant association of the change in SUVmax and progression-free survival observed for the least responsive tumor focus [22]. These studies underline the applicability and clinical significance of ^18^F–FDG-based hybrid imaging for therapy monitoring in melanoma under MAPK pathway inhibition. Providing a surrogate of tumor cellularity, DW-MRI confirmed the ^18^F–FDG-PET results and may thus be a suitable imaging modality to allow for a multi-facetted tumor characterization under targeted therapy. In experimental human BRAF-mutant melanomas, DW-MRI was successfully used for the *in vivo* monitoring of a 4-day therapy with the MEK inhibitor selumetinib [17]. Our results confirm the applicability of both ^18^F–FDG-PET and DW-MRI for therapy monitoring in the same tumors showing a strong, intraindividual correlation between ∆TTL and ∆ADC. This correlation may be explained by the fact that a high tumor cell density leads to restricted diffusion with low ADC values and increased tumor glucose metabolism [23].

The addition of a MEK inhibitor to BRAF inhibitor monotherapy proved to be a successful strategy to overcome intrinsic or de novo BRAF inhibitor resistance in BRAF-mutant melanoma [24]. However, resistance was also reported for the BRAF/MEK inhibitor combination therapy [25]. Therefore, novel compounds to be used in combination with BRAF inhibitors are required to address this clinical challenge and need to be investigated in experimental and clinical studies. As a proof of principle, the present study is a first approach to close this gap of knowledge, exploring a selective CDK 4/6 inhibitor as novel combination compound for dual inhibition of the MAPK signal pathway. Our preclinical results add to the literature providing first evidence that a novel BRAF and CDK 4/6 inhibitor combination therapy may be an effective therapeutic regimen in BRAF-mutant melanoma, with significant effects on tumor glucose metabolism, tumor diffusivity, microvascular density, and tumor cell proliferation. However, these promising preclinical results require further validation in future clinical studies. Selective CDK 4/6 inhibition was shown to significantly improve progression-free survival in patients with advanced breast cancer, but to date there are no clinical data on combined BRAF and CDK 4/6 inhibition [11, 26]. Our results indicate that ^18^F–FDG-PET/CT and DW-MRI may be applied for non-invasive therapy guidance in clinical trials investigating targeted MAPK pathway inhibition, adding functional and molecular biomarkers of therapy response to morphology-based response criteria. Measurements of tumor radiotracer uptake and ADC can easily be implemented in clinical routine staging reports. Clinically, ^18^F–FDG-PET/CT is widely applied for staging in advanced melanoma. The current European Society for Medical Oncology guidelines recommend CT or ^18^F–FDG-PET in advanced cutaneous melanoma (> pT3a) prior to surgical treatment and sentinel lymph node biopsy [27]. The National Comprehensive Cancer Network Clinical Practice Guidelines in Oncology for Melanoma Version 01.2017 recommend CT and/or ^18^F–FDG-PET for treatment response assessment in patients receiving active non-surgical treatment (www.nccn.org). While ^18^F–FDG-PET/CT provides a comprehensive whole-body tumor staging at a high spatial resolution, MRI is mainly applied for the local staging of defined body regions, e. g., for the detection of brain and liver metastases, and may therefore complement whole-body imaging protocols for the longitudinal assessment of therapy response [28]. State-of-the-art DW-MRI sequences, e. g., for the brain or abdomen, can be acquired in less than 3 min and may be integrated in standard MRI protocols without significant prolongation of the acquisition time.

### Limitations

The study is limited in several aspects. First, although all efforts were made to maintain the same scanning position between PET and MRI as well as between baseline and follow-up, it cannot be fully excluded that the animals were scanned in slightly different planes with potential effects on the semiquantitative parameters. Hybrid imaging on an integrated PET/MRI scanner with simultaneous acquisition of both modalities may improve consistency and concordance of the PET and MRI results due to equalized scanning conditions. Second, *ex vivo* immunohistochemical analysis was performed in selected cross-sections of the tumor and may not necessarily correspond to the whole-tumor VOI. Third, we investigated the BRAF and CDK 4/6 inhibitor combination therapy over the course of 1 week but did not compare the combination therapy to the BRAF inhibitor monotherapy. The comparison of the different therapy regimens in the same tumor model and a longer follow-up interval may provide additional insights in tumor pathophysiology and time course of acquired resistance under MAPK pathway inhibition. Fourth, due to study design, immunohistochemical parameters were only acquired on day 7 after follow-up imaging.

## Conclusion

In conclusion, we demonstrated that a novel BRAF and CDK 4/6 inhibitor combination therapy exhibits significant anti-angiogenic and anti-proliferative effects in experimental human melanomas in mice and that the according alterations in tumor pathophysiology can be monitored non-invasively and *in vivo* by ^18^F–FDG-PET/CT and DW-MRI. Co-targeting the MAPK pathway using a BRAF and CDK 4/6 inhibitor combination therapy is a novel approach to potentially overcome intrinsic or de novo BRAF inhibitor resistance in BRAF-mutant melanoma and may be investigated in future clinical trials.

## Abbreviations

^18^F–FDG-PET: ^18^F–Fluorodeoxyglucose positron emission tomography
ADC: Apparent diffusion coefficient
BRAF: B-rapidly accelerated fibrosarcoma
CDK: Cyclin-dependent kinase
CT: Computed tomography
DW-MRI: Diffusion-weighted magnetic resonance imaging
MAPK: Mitogen-activated protein kinase
MEK: Mitogen-activated extracellular signal-regulated kinase
MTV: Metabolic tumor volume
PMI: Platform for research in medical imaging
RECIST: Response evaluation criteria in solid tumors
SUV: Standardized uptake value
TTL: Tumor-to-Liver-Ratio
VOI: Volume of interest
